# Editorial: Additive manufacturing and biomaterials in regenerative dentistry

**DOI:** 10.3389/fbioe.2023.1211009

**Published:** 2023-05-10

**Authors:** Monireh Kouhi, Mohammad Khodaei

**Affiliations:** ^1^ Dental Materials Research Center, Dental Research Institute, School of Dentistry, Isfahan University of Medical Sciences, Isfahan, Iran; ^2^ Materials Engineering Group, Golpayegan College of Engineering, Isfahan University of Technology, Golpayegan, Isfahan, Iran

**Keywords:** additive manucatruing, biomaterials, dentistry, tissue regeneration, titanium based bioimplants

Clinical approach to heal hard and soft tissue defects in dental and maxillofacial area sometimes rely upon biomaterials based mass products fabricated for a variety of surgical treatments, however, additive manufacturing and 3D printing express a paradigm shift in producing patient specific medical devices. Additive manufacturing or 3D printing are a collection of technologies allowing the fabrication of 3D structures via a computer and a computer aided design (CAD). Printing materials or ink can include metals, polymers, ceramics and cells. When cells are included in the printing ink, the technique is called 3D bioprinting which enables the precise deposition of cells, matrix, and signaling factors to produce sophisticated objects. It has the potential to revolutionize the dental treatments and to speed up transition from traditional restoration approach towards bioengineered solutions. Regarding this, Ostrovidov et al. (Frontiers in Bioengineering and Biotechnology, 2023, 11, 991821) reviewed the recent advances in different bioprinting techniques (such as extrusion based, stereolithography, inkjet, laser assisted) and bioinks for application in regeneration of different dental alveolar tissues including dentine, dental pulp, bone, periodontal ligament, etc. The bioprinting regulation for both 3D printed constructs, without cells (regulated as medical devices) and with cells (regulated as biologics and drugs), is also discussed in this review paper.

One of the challenges in the reconstruction of oral and maxillofacial defects is available biomaterials, which may not be appropriately able to perform true formation of new tissues. Optimization and fabrication of bioresorbable scaffolds with the capacity to reconstruct defects reliably and safely must be the major focuses of the future researches. Further, the potential functionalization of scaffolds for therapeutic purposes should be consider when designing regenerative bio constructs.

Most of the available bone grafting materials in clinics lack appropriate degradability and efficient osteoinductivity. In a study by Xu et al., a novel biomimetic nanocrystalline calcium phosphate containing bone morphogenetic protein-2 (BMP-2) was developed to prepare degradable and highly osteoinductive granules to heal critical sized bone defects. The results of cytotoxicity and cytocompatibility assessment of MC3T3-E1 pre-osteoblasts showed no obvious cytotoxicity and improved pre-osteoblast cells adhesion. *In-vivo* histomorphometric analysis revealed that the new bone volume induced by the developed granules showed a BMP-2 amount-dependent increasing behavior.

Titanium and its alloys are being widely used for bone repair purposes, due to their high biocompatibility and strength/weight ratio. (Su et al.), compared the properties of titanium with other dental implant materials such as zirconia and polyetheretherketone (PEEK). They stated that titanium had lowest water contact angle and better wettability, higher cell viability and improved alkaline phosphatase activity compared to other studied materials. The most interesting matter is that, the anti-adhesion effect against bacteria of titanium was significantly better than zirconia and PEEK.

Besides lots of benefits, titanium is inherently bioinert and cannot bond to surrounding tissue after implantation. To alleviate this restriction of titanium, many different surface treatment techniques have been used by researchers to improve titanium bioactivity and osteoconductivity. Regarding this, in a review paper by Nansi López-Valverde et al., chitosan coating on the surface of titanium implant was mentioned to enhance antibacterial ability, osteoinductivity and osseointegration capability of titanium. The superior biological properties of chitosan and also its excellent ability to bond on the surface of titanium, can lead to applying chitosan coating on the surface of titanium dental implants in future. Different techniques can be used for chitosan coating on the surface of titanium implants.

Besides the coating technique, acid etching can be used to improve the surface properties of titanium. Yan et al. 3D printed Cu-bearing titanium alloys and applied acid etching on the surface of 3D printed titanium alloys, they concluded that biological properties of the Cu-bearing titanium alloys could be significantly improved upon etching treatment with acid. Acid etching treatment could enhance cell adhesion, activate macrophage polarization, and reduce ROS levels on the surface of titanium.

Superior properties of titanium and also lots of surface treatment techniques resulted in widely implantation for dental and bone repair. However, not only titanium and its alloys, but also titanium oxide (TiO_2_) in the forms of anatase and rutile are biocompatible and also are bioactive. So, titanium oxide nano particles are widely used as reinforcement (filler) of nano-biocomposites and also as coating on the surface of implants. Yu Ma et al. used titanium oxide nano particles to reinforce glass ionomer cement, it was reported that titanium oxide addition to the glass ionomer cement resulted in mechanical properties improvement, significantly. The strength, hardness and antibacterial properties and also bioactivity of anatase and rutile (two different forms of titanium oxide) lead to clinical wide application of titanium oxide nano particles.

Beside the treatment of hard tissue defects, the soft tissue regeneration and wound healing strategies are of great importance in oral and maxillofacial area and remains a significant clinical problem due to inflammation, infection, and dysangiogenesis. A variety of biocompatible materials in the form of hydrogels, membrane and lubricants have been investigated to enhance wound healing rate. Regarding this, Lu et al., developed a novel injectable hydrogel based on catechol modified chitosan and aldehyde modified cellulose modified nanocrystal containing Ag. The developed hydrogels showed *in situ* forming properties, suitable mechanical properties, biocompatibility, controlled release of Ag, and antibacterial performance, as well as promoted neovascularization and tissue regeneration. Additionally, the obtained hydrogel was able to completely cover and firmly attach wounds with irregular shapes, so eliminates the re-injury process. In another study, a water-based polyethylene glycole (PEG)/chitosan (CS) composite lubricant was successfully developed for wound healing (Gao et al.). The L929 cells viability test indicated the good biocompatibility of the PEG/CS lubricant, and the results of tribological test revealed that it had a good lubrication effect. In addition, the developed PEG/CS lubricant has been proved to possess a good wound-healing effect as resulted from the animal study.

Biocompatible materials are also applied in dentistry field as desensitization materials to effectively alleviate dentin hypersensitivity (by occluding exposed dentinal tubules), and inhibit S. mutans to prevent caries simultaneously. For instance, in a study by Yan et al. an approach for the development of IgY-loaded amorphous calcium phosphate (ACP) (IgY@ACP) has been proposed, which have synergetic advantages of IgY and ACP in enabling the dentin surface to resist dentin hypersensitivity and caries. Their results showed that IgY@ACP could stably occlude dentinal tubules with acid challenge and effectively inhibit the formation and growth of biofilm of S. mutans and could be a promising desensitization material for dentin hypersensitivity therapy.

